# Zinc Dies for Swaging Plates

**Published:** 1883-08

**Authors:** Wm. H. Dorrance


ARTICLE II.

### ZINC DIES FOR SWAGING PLATES.

BY WM. H. DORRANCE, D. D. S.

"Come now, and let us reason together."
"The proof of the pudding is in chewing the string."

In the March number of the *Ohio State Jonrnal* appears a short article entitled "About Impressions and Other Things," in which the writer; a dentist of "thirty-seven years practice," offers to students and others who have been instructed "in annoying and uncertain methods," "a few helpful thoughts," interlarded with some that are not helpful, and seasoned with running criticisms on "uncertain methods."

In this article its writer reiterates the advocacy of the exclusive use of a non-shrinking alloy for "dental dies," without, however, offering any argument in favor of such exclusive use, other than the fact that he has used it for thirty years.

It is therefore the purpose of this paper to state a few facts (the partial result of experiments now in progress,) and give a few reasons, by way of argument against the habitual use of non-shrinkable dies. And, inasmuch as the above-mentioned writer makes some blunders in the written and implied criticisms on methods, as he says, "written and taught in dental journals and colleges, or some of them." It is also proposed to examine those criticisms in a critical way, and, in a running commentary, to offer suggestions of methods that will appeal to the reason of those interested in metal work.

The difficulties in the practice of swaging plates are not so much due to the inherent unmanagebleness of the materials used, as to a lack of skill on the part of the operator. It is doubtless safe to say that ninty-five per cent. of the dentists of to-day are entirely unacquainted with the practice of swaged plates, and it is equally true that the five per cent. who are, per contra, practically familar with the work, are so from long practice. No one, however well equipped naturally, can be an expert in the real art and science of dentistry, in any of its branches, in a few short years, and for the many, a lifetime is all but too short to reach the highest point of excellence. This is especially true in prosthetic dentistry, and it is not so much "in consequence of the annoying and uncertain methods in which they have been instructed" that makes men "stick to rubber work," as it is due to the lack of the practical ability that comes from long habit, and the skillful training of the faculties and fingers. Now, theories, though "fine spun," when backed with facts, becomes principles, and principles are worth investigation, and before considering the practice of swaging plates, it will be well to state the following fundamental principles:

An upper plate is perfectly adapted only when it rests with equal bearing upon the surface and structures which it covers, which surface is invariably unequal in its density and consequently in its resistance to pressure, when pressure upon any point of its lingual or buccal surface fails to dislodge it.

These principles are relatively true in regard to the lower plate, it may also be stated, as another axiom, that even the best plaster model represents the mouth only in one particular—that of approximate shape, as it is impossible with a material of equal resistance throughout its bulk to represent a surface of unequal density and resistance.

Hydrated calcium sulphate, of good quality, and properly prepared for taking an impression, will, from the moment crystallization begins until its completion, expand in the ratio of one two-hundredth of its bulk, in other words, the co-efficient of expansion is .005. That is to say, an impression of good plaster in its best condition, which has a breadth of two inches, has expanded one-hundredth of an inch. If the same quality of plaster is carelessly prepared —as we may claim a large proportion of it is—the co-efficient of expansion will run as high as .01 (and even .02 in some cases,) or, in an impression of a breadth of two inches, will be increased one-fiftieth of an inch. If then the best of care is taken, with the impression and the resulting model, the latter which has the width of two inches, is larger than the mouth which is supposed to represent by one-fiftieth of an inch, and may be larger by one twenty-fifth of an inch, or even more. Should the varnish used to protect the cast from moisture and injury be too freely applied, the cast is still further enlarged, and though it is reasonable to suppose that the process of moulding would leave the mould slightly larger than the model, it will not be taken into account in this connection. Zinc, in being raised from 75 to 773° F., has a co-efficient of expansion of .011, but has this noticeable peculiarity, viz: Its contraction on cooling from the fusing point to, say 75°, is not equal to its previous expansion, especially is this noticeable in the case of the cast in the open mould, where the contraction is greater in the center of the exposed upper part and diminishes somewhat to a point not quite reaching the lower surface. Let a case be properly taken through the steps of manipulation from the plaster model to the zinc die, and the die will be found to be, for two inches on the model, one seventy-fifth of an inch less than the model, the shrinkage of the zinc doubtless being partly compensated by the slightly increased size of the mold over the model.

An alloy of one part copper, two parts antimony, and eight parts tin, will be found to have a co-efficient of expansion in *cooling* from fusion to 75° of .003, nearly, and a die of that alloy, under like circumstances, with the zinc die, will be found to be one two-hundredths of an inch larger than the model, and consequently larger than the month,

(by its own expansion *plus* the expansion of the plaster,) whereas, the zinc die is about that much smaller than the mouth, and with very infrequent exceptions, this proves an exceedingly desirable difference, the slight compression of the soft tissue materially aiding in the stability of the plate. It is notorious that the mouth adapts itself to adverse conditions, and instances are innumerable of the wearing of wretchedly illfitting plates, which are retained by the ready adaptation of the soft tissues covered by it, and the use of the muscles of the face, but are clattered about by every action, and are ready to fall at the slightest touch. Serious injury to the mucous membrane and gums is the result of wearing such dentures, and with the lapse of time the injury increases. No one with any desire to serve humanity to the best of his ability, can look with complacency on such dentures, or wish to see the number increased.

Although the adaptation of a plate approaches perfection in the ratio of the perfection of the impression, it is the exception rather than the rule, that the plate requires no change in fitting it to the mouth, or cannot be benefited in some respect, and in the swaged plate, the intelligent skill of the expert manipulator is afforded abundant swing in the matter of fitting. Quite a little toward securing perfect adaptation may be accomplished in the handling of the impression and model, but it will quite invariably be found that when the plate is perfectly adapted to the mouth, it will no longer even go upon the zinc die, much less the plaster model from which it was obtained, and any attempt to force it upon either of them should not there be made. But when once it is perfectly adapted, the reasonable and safe course is to prepare a model from the plate, to be kept for future reference, and one will meet with the proof of the slight expansion of the plaster, in the removal of this cast from the plate. Now the alloy known as babbitt metal has its use to serve, and should be in the hands of all who would do metal work, so, too, there are other materials which have their place, as tin, plumber's solder, brass, etc.

One, to do well, should choose from the materials at command that one or those which will best serve the end. Once in a while it will be found that the plate swaged on the zinc die is somewhat too small, and then the unshrinkable alloy becomes useful.

In examining the criticisms referred to at the beginning of this article, let some of them be taken in order.

The writer says: "To say that plaster will not make a sharper impression than wax or modeling compound, is arrant nonsense;" and without knowing whom he means to hit in that sentence, and while agreeing in the main with the statement, it is well to say that those who are familar with the use of the impression materials mentioned, viz: wax and modeling compound, (composition magnifique being by far the finest of the compounds,) together with others of the same type, regard them as equally valuable in their especial province and uses as plaster in its uses. The balance of the paragraph, however, demands notice: "And to compel a student in college to spend weeks of "valuable time in learning to take wax impressions, (and I "have ' known some such, who, after graduating, could not "take a plaster ' impression successfully!) is an imposition "that ought not be ' tolerated." The student or practitioner who, even at the expense of "weeks of valuable time," can uniformly secure a good impression with wax, modeling compound and gutta-percha, not only is in possession of a valuable acquirement, but is much better prepared to take a good plaster impression. And furthermore, those in the college doubtless referred to, who "could not take a plaster impression successfully," were also unable to take a good impression in wax, and will probably never be successful in any operation involving skillful manipulation. Moreover, the criticiser can be shown models made from wax, modeling compound, and plaster impressions, the work of students who three months previously had never seen an impression, the equal of which would bother nine out of ten dentists, as they run, to produce.

In the next paragraph he says: "Hence again come in nonsensical theories about the use of shellac and oil in filling the impression, etc." Those who have acquired the habit of separating the impression and model with water alone, and are familar with the elegant results, will laugh at the statement of the "thirty-seven years" practitioner who says, "the thin coat of oil is equally unobjectionable," and will not be induced to give up the beautiful, sharp and clean surface of the water-separated model for the one blurred by the use of oil. The thin shellac he speaks of is well enough in difficult cases, provided it is thin, when it answers as well as any other coloring to show the line of separation; but how often, instead of thin shellac, it is thick shellac, and becomes a coating instead of a marking, to the detriment of the model.

In the succeeding paragraph, after speaking with an exclamation point of the method of overlaying the die with lead, foil or sheet, to relieve pressure, he says: "The method ought to be patented." Then follows a curious "sentence: scrape the "rear of the cast slightly between "the coronoid process and near the center, from where the "edge of the plate would come, forward one-quarter of an "inch." Why not scrape it between the occipital protuberance "and near to the center," and have that method patented? It is to be supposed that he refers to the maxillary tuberosity.

It is true that oiled sand is "always ready for use, not "needing to be renewed for weeks or months," but is also true that this particular advantage is more than counterbalanced by the penetrating odor of the burning oil. Tastes differ in regard to smells—the most people preferring something less pungent. And then he says: "In view of such results," (what results?) "it is a pity that "students should be compelled to devote so much precious "time to the making of zinc dies, a method that is perplex-"ing, slow, and uncertain in results, and which should, long "ago, have become obsolete." Does he mean to convey the idea that there is any possible difference in the manipulation of zinc and the alloy he advocates, save that of the somewhat increased melting point of the zinc? Certainly in every other respect zinc has the advantage.

It is to be presumed that it is on the score of economy of expense of apparatus (rather than time) that he advises: "if the case is badly undercut, make a 'core,' thereby "avoiding in a simple (!) way the necessity of using the " 'Hawes' flask, as he would find it difficult to induce anyone who is in the possession of that elegant flask (though its cost is ludicrously high,) to take the roundabout way of coring an upper model. Our critic will possibly mislead "some when he says, 'if careful to wipe off any base "metal that may adhere . . . . . there will be no need of putting the plate in acid before annealing," as it is not always possible to wipe off the base metals from the oiled plate, and one might better err on the side of safety and use the acid both before and after annealing. The best work is produced when the plate, at all stages of the work, is kept not only clean, but also with a surface free from scratches and marks, and even well polished.

The next paragraph gives comfort to the lazy man, and very poor advice to anyone, together with two grave errors. He says: "Don't hesitate to cut your full upper plate in "front and lap it, for while it saves times and annoyance, it "also increases the strength of the plate at the point where "there is the most "strain,"—error one: for the strain is not a pulling strain upon the lap, but a pushing strain, which would be more likely to buckle the plate on either "side of the lap. And, "there is far less danger of the "plate warping than if it has been cramped into an unnatural condition by swaging without cutting;"—error two: for if the plate has been kept properly annealed the shape is no more unnatural, as far as the disposition of its molecules is concerned, than at the start, and consequently no more likely to warp in soldering, if the heart is properly applied.

The closing paragraph of his article also needs notice in several points. A rather slip-shod (though sometimes admissable) method of backing up teeth is advised as the rule of work, as it " saves time " and is said to be " just as good " as better methods. Though skill and long practice will do much, it is reasonable that no one can so well shape, fit and adapt a backing to a plate tooth when it is hidden by the investment and adjoining teeth, as before it is invested, neither can it be controverted that the work can just as expediously done before investment. No wonder he advises to " invest in plaster and sand," which he says " is preferable to asbestos, because it is not so yielding in backing up," and " in a sheet-iron ring " to hold his work together! He has in that sentence stated the only possible objection to asbestos, but if he will discard his plaster and sand, and intelligently use plaster and asbestos instead, he will soon find it to possess the following immense advantages: in equal bulk with the sand investment it will require less heat to do the work; the investment may, and should be much thinner; the sheet-iron ring will be utterly superfluous, for with the proper proportions of plaster and asbestos, (which are easily determined) the investment will not change, warp or crack.

To those who are not familar with this investment, the following method of determining the proportions will be valuable as a guide. In a sufficient quantity of tepid water dissolve from three to ten grains of potassium sulphate, using more with hard water than with soft water; add the asbestos (short fibers preferably) and stir until it becomes saturated, pour off the surplus water, should there be any, and add plaster until it is as thick as can be conveniently handled.

But to return to the paragraph under examination: The writer tells us to " use thick gold, say about guage 24 "— and as he does not tell us whose guage, we will suppose it to be Brown & Sharp's U. S. Standard. Now, plate of that guage is about the right thickness for an ordinary plate, and certainly the backing should be much thicker. It only remains for one to wonder why one needs to "burr off the heads of the pins," after soldering, when one has previously "cut off the surplus with a sharp tool."

March 29, 1883.

